# Metagenomic insights into effects of wheat straw compost fertiliser application on microbial community composition and function in tobacco rhizosphere soil

**DOI:** 10.1038/s41598-019-42667-z

**Published:** 2019-04-16

**Authors:** Yongfeng Yang, Songjie Zhang, Ning Li, Hongli Chen, Hongfang Jia, Xiaoning Song, Guoshun Liu, Chao Ni, Zhizhong Wang, Huifang Shao, Songtao Zhang

**Affiliations:** 1 Henan Agricultural University, College of Tobacco Science, Tobacco Cultivation Key Laboratory of China Tobacco, Zhengzhou, 450002 China; 2 China Tobacco Chongqing Industrial Corporation, Chongqing, 400000 China; 3 Wuyang County Tobacco Branch of Luohe Tobacco Company, Luohe, Henan Province 462000 China

## Abstract

The application of fertilisers incorporated with plant residues improves nutrient availability in soils, which shifts the microbial community structure and favours plant growth. To understand the impact of wheat straw compost fertiliser on soil properties and microbial community structure, tobacco planting soils were treated with four different fertilisers using varied amounts of straw compost fertiliser and a no fertiliser control (CK). Results showed that different fertilisers affected available soil nutrient contents differently. Treatment of tobacco soil with application of combined chemical fertiliser/wheat straw compost led to improved soil chemical properties, and increased soil organic matter and available phosphorus and potassium content. Treatment with FT1 200 kg/mu straw was found to be superior in improving soil fertility. Metagenomic DNA sequencing revealed that different fertiliser treatments resulted in changes in the microbial community composition. In soil treated with FT2 300 kg/mu straw for 60 days, the predominant bacterial phyla were Proteobacteria, Actinobacteria, and Verrucomicrobia, whereas Cyanobacteria, Basidiomycota, and Chlorophyta were found in high abundance in soil samples treated with FT1 200 kg/mu straw for 30 days. Functional annotation of metagenomic sequences revealed that genes involved in metabolic pathways were among the most abundant type. PCoA analysis clearly separated the samples containing straw compost fertiliser and chemical fertiliser. A significant correlation between soil properties and the dominant phyla was identified.

## Introduction

Environmental soil degradation and production sustainability in different agricultural systems have aroused public concern about soil fertility and quality^1^. As previous studies have shown favourable effects of soil nutrients (e.g. organic C, N, P and K) on the physical, chemical, and biological properties of soil, concentrations of these soil nutrients are good indicators of soil quality and productivity^2^. Long-term continuous cropping leads to lack of carbon in the soil, leading to an imbalance in carbon and nitrogen and a reduction in soil nutrient availability^3^. With the development of agricultural production, appropriate fertilisation has been widely used as an important management practice for maintaining soil fertility and quality^4^ and improving crop yields^5,6^. The long-term overuse of chemical fertilisers induces soil compaction and acidification in tobacco planting soils^3,7^. Therefore, organic and inorganic amendments, including application of nitrogen fertilisers in combination with straw are returning as recommended approaches to increase the availability of nutrients and improve the yield and quality of plants, including tobacco^8^.

Previous studies have shown that different fertilisation strategies influence carbon cycling in soil^9,10^, and therefore, influence the phylogenetic structure of the soil microbial community^11^. Results from previous studies have shown that nitrogen amendments affect a wide range of bacterial^12^ and fungal communities^13^. Organic and inorganic fertiliser amendments can also affect the composition of soil microbial communities^14,15^. These shifts in community composition are likely associated with changes in the functional capabilities of the communities as well. Moreover, a recent laboratory-based study^16^ supports the concept that soil microbial communities are neither functionally redundant nor similar. Recent research has demonstrated that prolonged elevations in nitrogen availability may directly or indirectly change the microbial carbon dynamics, thereby shifting the composition of soil microbial communities, their catabolic capabilities, and metagenomes^17^.

A recent study indicated that the application of straw, with and without straw decomposer, could shift the soil bacterial community structure, specifically activating the copiotrophic bacteria and increasing the soil biological activity, thus, contributing to soil productivity and sustainability in agro-ecosystems^6^. Increasing number of research studies have demonstrated that chemical fertilisers with straw significantly enhance bacterial abundance^18,19^. Recent studies have demonstrated metagenomic, phylogenetic, and physiological responses of microbial communities in soil^17^. Indeed, advances in next-generation DNA sequencing approaches combined with traditional microbiological and chemical analyses of soil parameters may provide a biologically relevant assay to assess the potential effects of fertiliser applications and straw compost amendments on soil microbial communities.

Thus, in this study, we investigated the impact of different fertiliser treatments on microbial communities in tobacco planting soils. Soil samples were collected from a long-term field experiment involving use of conventional fertiliser treatments alone or in combination with wheat straw compost. Tobacco traits and soil properties were analysed and a metagenomics sequencing approach was undertaken to examine the microbiological profile diversity.

## Results

### Effects of different fertiliser treatments on soil fertility and physicochemical properties in tobacco field soils

**Figure 1:**
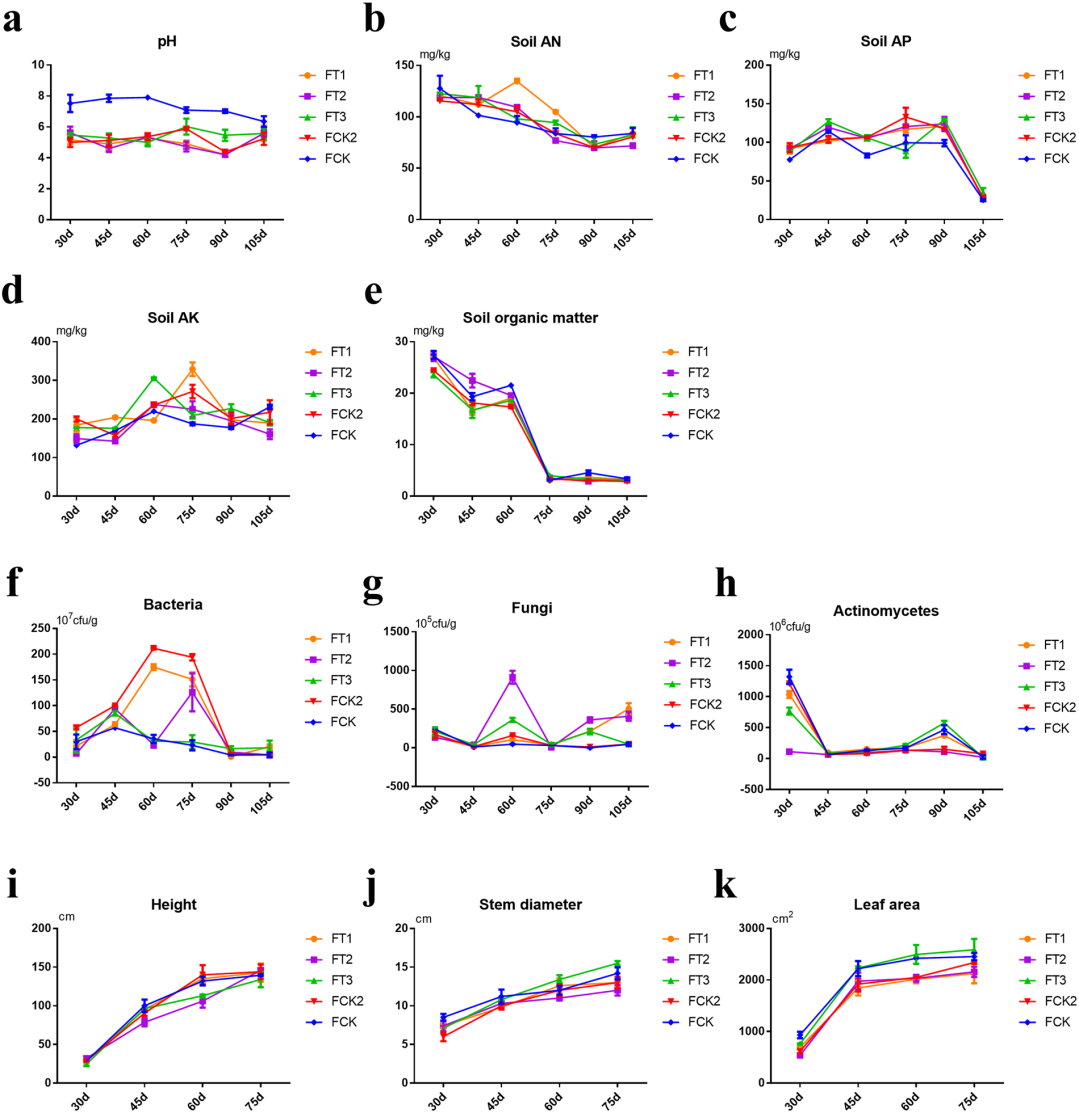
Soil properties under different fertiliser treatments. (**a**) Soil pH, (**b**) Alkaline nitrogen (AN) in soil, (**c**) Available phosphorus (AP) in soil, (**d**) Available potassium (AK) in soil, and (**e**) Organic matter in soil. Effects of different fertiliser treatments on microbial populations: (**f**) Bacteria, (**g**) Fungi, and (**h**) Actinomycetes. Agronomic characteristics of tobacco under different fertiliser treatments: (**i**) Height, (**j**) Stem diameter, and (**k**) Leaf area.

Chemical and physical properties of soil were measured after fertiliser treatments. Soils with pH 5.5–6.5 are considered suitable for tobacco planting and high-quality tobacco leaf growth. Our results showed that the application of wheat straw compost fertiliser had no evident effect on the soil pH, which remained at about 5.7 (Fig. 1a). Available N content in the soil was decreased with tobacco growth periods, which were regained at 60 days post-transplantation with the FT1 200 kg/mu straw treatment (Fig. 1b). Available phosphorus content continued to increase, reaching a peak at 90 days post-transplantation, which was evident in the samples that underwent FT3 400 kg/mu straw treatment (Fig. 1c). There were no evident changes in available potassium content with wheat straw compost fertilisers. However, available potassium content was significantly increased by treatment with FT3 400 kg/mu straw at 60 days post-transplantation (P < 0.001) and FT1 at 75 days post-transplantation (P < 0.001) (Fig. 1d). Organic matter content in the soil remained unaltered during the growth period before 60 days post-transplantation. The organic matter content of the different soil samples was in the following order: FT2 300 kg/mu straw > FT3 400 kg/mu straw > FT1 200 kg/mu straw (Fig. 1e).

Initial increase observed in the bacterial population in the rhizosphere soil of the tobacco field was decreased later. The application of FT1 200 kg/mu straw and FCK2 pure nitrogen led to a significant increase in the bacterial population 60 and 75 days post-transplantation (P < 0.001, Fig. 1f). Post-transplantation bacterial population with FT2 300 kg/mu straw treatment showed increase at 45 days, decrease at 60 days and reached peak at 75 days. With FT3 400 kg/mu straw treatment, the bacterial population reached a peak at 45 days post-transplantation followed by a gradual decrease. The fungal population showed increase with an increase in wheat straw compost and reached a peak at 60 days post-transplantation (Fig. 1g), indicating that wheat straw compost fertiliser, especially FT2 300 kg/mu straw, could increase the fungal population. The population of actinomycetes reached a peak at 30 days post-transplantation, then decreased at 45, 60 and 75 days, and again increased at 90days (Fig. 1h), indicating direct or indirect increase in the population of actinomycetes through the application of wheat straw compost fertiliser, especially FT3 400 kg/mu straw, at the later stages of tobacco leaf growth.

Significant changes in microbial population occurred mainly after 30, 60, and 90 days treatment with 4 fertilisers. Also, these three time points were mapped to growth stages of tobacco. Therefore, the plants at 30, 60, and 90 days after treatment with each fertiliser were chosen for metagenome sequencing.

### Effects of different fertiliser treatments on tobacco traits and properties

Agronomic traits were assessed during various tobacco growth stages after transplanting with different fertiliser applications. The difference between the height and the growth rate of the tobacco plant was maintained at a static rate, but the maximum leaf area per stem and the maximum leaf area per plant was greater under the FT3 400 kg/mu straw treatment (Fig. 1i–k). Regarding factors of botanical traits, disease index and appearance quality of the original tobacco, treatments with wheat straw compost fertiliser showed superiority in all areas. Botanical traits were improved, the incidence of mosaic disease and black shank were reduced, and the applications of FT1 200 kg/mu straw and FT3 400 kg/mu straw led to superior gloss and oil content of tobacco leaves. However, in terms of agronomic properties and economic traits, tobacco grew better under the treatment of FT3 400 kg/mu straw than under other treatments, but showed the highest incidence of black shank disease. Overall, the application of FT1 200 kg/mu straw showed superiority for all the traits. Moreover, the contents of total sugars, reducing sugars, nicotine, chloride, potassium, and other nutrients were also determined in the tobacco samples under different fertiliser treatments (See Supplementary Table S1–S5).

### Metagenome sequencing and characteristics under different fertiliser treatments

To further compare microbiomes, metagenomic sequencing was carried out for these soil samples using the Illumina platform. Numerical data for metagenomic sequencing and assembled scaffolds are summarised in Table 1. After annotation, numerical data is shown in Supplementary Table S6. At the domain level, genes affiliated with the bacterial domain were predominant at 64.1–78.5%, while archaea associated genes only represented a minor proportion of the microbial community at 1.2–4.7% (See Supplementary Table S7). In all samples, the Eukaryota domain accounted for 14.1–26.9%. The remaining identified genes comprised DNA viruses and unclassified organisms.

**Table 1:** Summary of numerical data for metagenomic analyses.

Sample	Total Reads	Total Bases	Scaffolds	Genome size(bp)
Sample FCK2 30d	48009382	7201407300	134419	53597709
Sample FCK2 60d	54397252	8159587800	177237	73237076
Sample FCK2 90d	65740222	9861033300	180434	73791045
Sample FT1 30d	56166616	8424992400	108429	44720269
Sample FT1 60d	58391596	8758739400	98228	32999316
Sample FT1 90d	56670216	8500532400	187021	76096525
Sample FT2 30d	53791180	8068677000	77643	30355554
Sample FT2 60d	46155948	6923392200	48439	15286015
Sample FT2 90d	58657420	8798613000	161384	59297494
Sample FT3 30d	54705762	8205864300	82462	32839885
Sample FT3 60d	47970042	7195506300	93715	32946042
Sample FT3 90d	52476706	7871505900	145542	63607372

**Figure 2:**
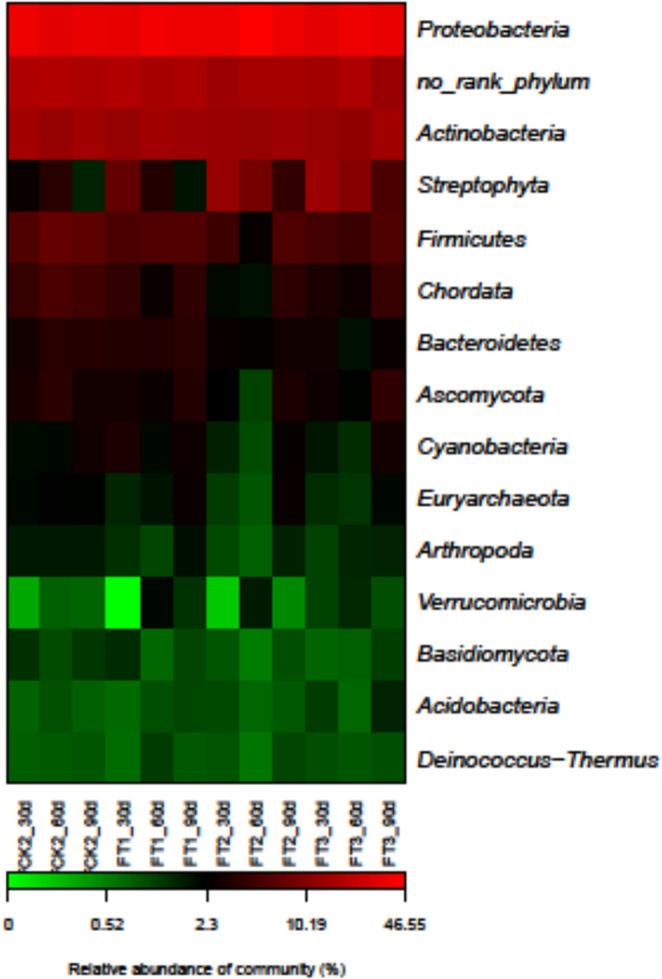
Heatmap of soil microbial community abundance profiles at the phylum level.

Taxonomic analysis showed that all metagenomic samples were dominated by phyla Proteobacteria, Actinobacteria, Firmicutes, Bacteroidetes, Streptophyta, and Ascomycota. The heat map of abundance profiles showed that some phyla were overrepresented in the wheat straw compost-based samples. For example, the relative abundances of phyla Proteobacteria, Actinobacteria, and Verrucomicrobia were high in the sample FT2 300 kg/mu straw 60-day, whereas high abundances of Cyanobacteria, Basidiomycota and Chlorophyta were observed in the FT1 200 kg/mu straw 30-day sample (Fig. 2).

### Taxonomic comparison of the sampled communities under different fertiliser treatments

We made further taxonomic comparisons using community abundance profiles in the four fertiliser treatments. The heat map results showed that the different fertiliser treatments influenced the abundance of microbial communities (See Supplementary Fig. S2). We also compared the microbial community abundance profiles across different fertiliser treatment samples (See Supplementary Fig. S3).

**Figure 3:**
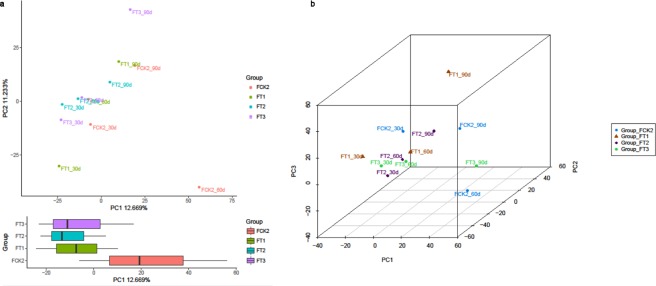
Principal component analysis (PCA) of microbial communities in 12 soil samples under the four treatment regimes (**a**). Principal coordinates analysis (PCoA) plot depicts the Bray-Curtis distances between microbial communities in the 12 soil samples under the four treatment regimes (**b**).

PCA was carried out on the relative order count data from the metagenomic reads. The greater the similarities in sample compositions, the closer were the distances in the PCA map. The results indicated that the soil under treatment of wheat straw compost fertilisers (FT1 200 kg/mu straw, FT2 300 kg/mu straw, and FT3 400 kg/mu straw) showed significant differences compared to soil amended with FCK2 pure nitrogen fertiliser (Fig. 3a). Samples from different groups showed a decentralised and aggregated distribution.

The PCoA analysis showed differences between individuals and groups. Each sample is represented by a coloured spot in the graph and the distances between the spots reflect the original distances between the samples which were calculated using the Bray-Curtis ecological index. Accordingly, PCoA analysis was performed for comparative purposes, which clearly divided the samples into well-separated populations (straw compost amendments vs. chemical fertiliser) (Fig. 3b) based on the composition of the microbiota.

### Functional comparison

**Figure 4:**
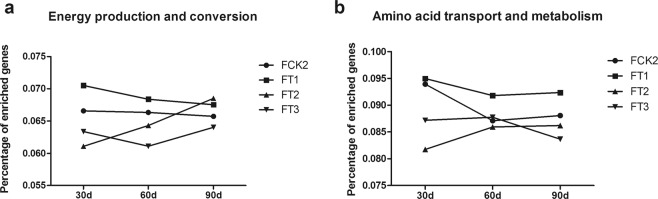
The time course-changes of gene abundance in energy production and conversion (**a**) and amino acid transport and metabolism (**b**).

In order to explore the function of metagenomes after treatment, the metagenomes were annotated against KEGG and COG databases. The genes of samples for each treatment at 30, 60, and 90 days were all significantly enriched in energy production and conversion, amino acid transport and metabolism (all p < 0.05). The two signal pathways showed significant differences in functional enrichment analysis among different treatments (all p < 0.05). The changes of the abundance of the two functions at different time points are shown in Fig. 4.

**Figure 5:**
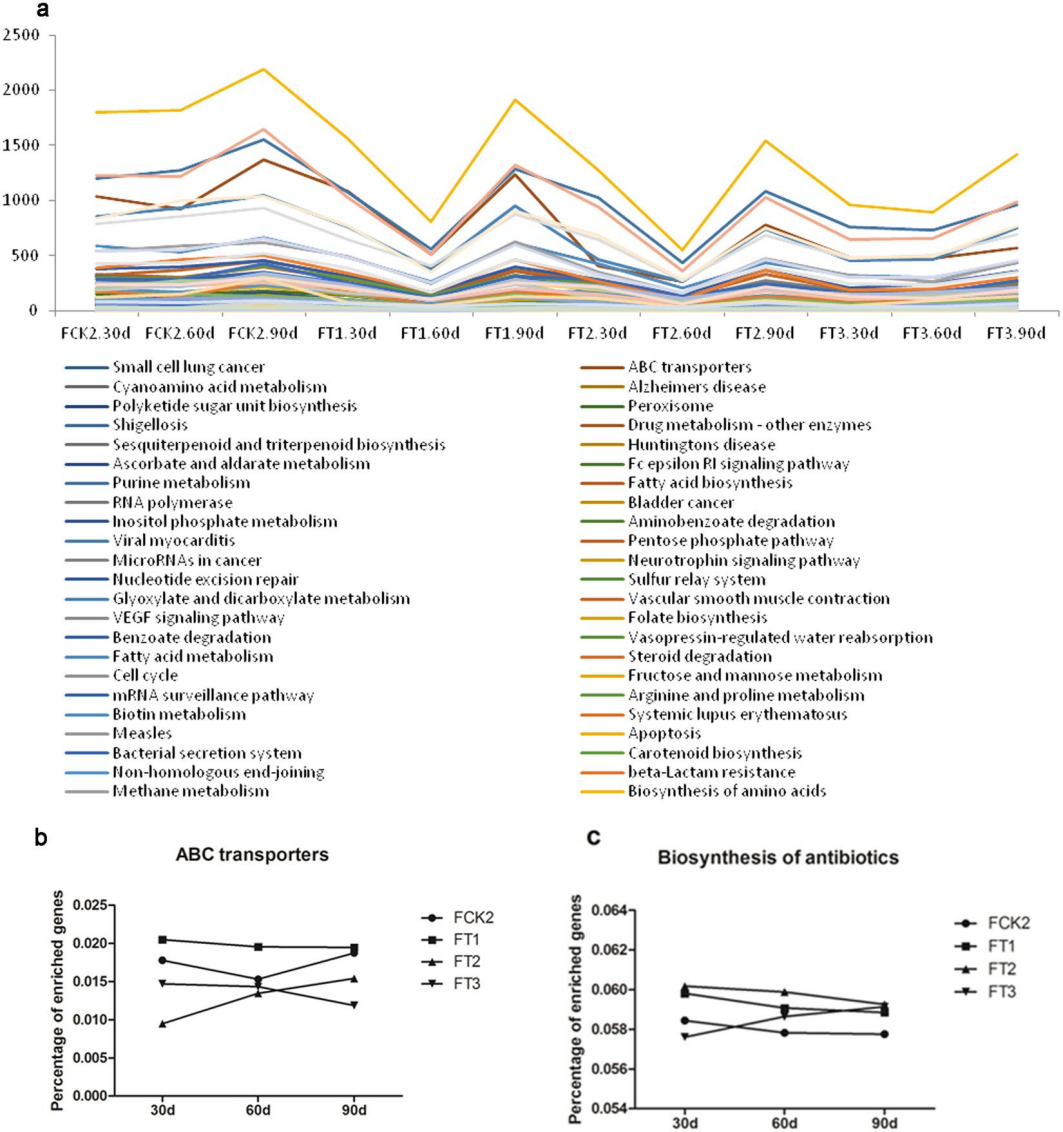
Abundance trends of Kyoto Encyclopedia of Genes and Genomes (KEGG) pathways (**a**). The time course–changes of gene abundance in ABC transporters and (**b**) biosynthesis of antibiotics (**c**).

In the KEGG functional analysis, the results showed that, in general, different groups of genes displayed a down-regulating trend at 60 days but showed an up-regulating trend at 90 days (Fig. 5a). Metabolism was among the most abundant pathways; moreover, biosynthesis of secondary metabolites, biosynthesis of antibiotics, microbial metabolism in diverse environments, and carbon metabolism also ranked as the most abundant gene pathways across all samples (See Supplementary Fig. S4). The variation that occurred between 60 days and 90 days was more pronounced in soils amended with wheat straw compost fertiliser. There were significant differences in the enrichment of ABC transporters (p = 0.0069) and biosynthesis of antibiotics (p = 0.0183) pathways among samples with different treatments. The temporal changes of the enrichment of the two pathways were showed in Fig. 5b and c.

### Linking soil properties with microbial communities

**Figure 6:**
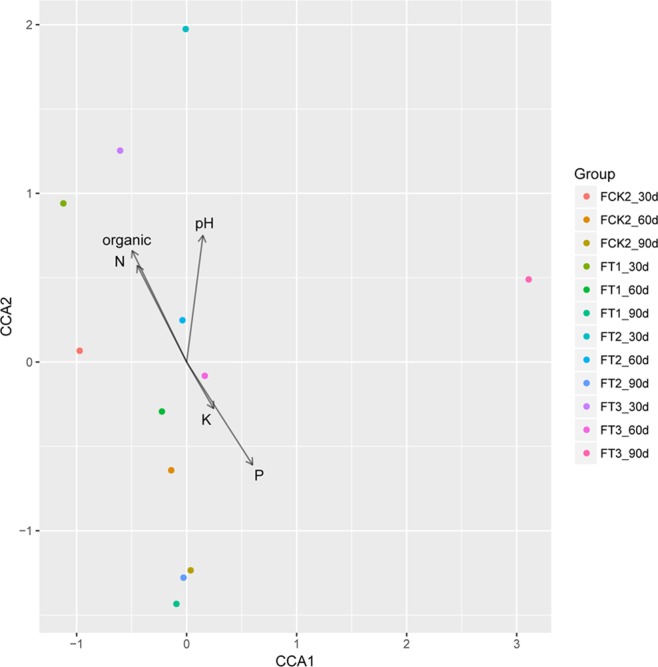
Redundancy analysis of abundant phyla and soil properties. The results showed that changes of pH and N content were significantly associated with the microbial communities, while the values of Pr > F were 0.044 and 0.010, respectively.

Redundancy analysis was performed to explore the relationships between microbial populations and soil properties. Considering the entire microbial community composition, our study revealed a significant correlation between soil properties and the dominant phyla identified (Fig. 6).

## Discussion

The crop yield and healthy food production is dependent on plant nutrients. The type of fertilisers used is essential for the increased production to provide sufficient nutrients for plants. The sound management of fertilisers is essential to ensure the plant production and environmental safety. In this study, we evaluated the effects of chemical fertiliser treatments with and without wheat straw compost fertiliser on microbial communities in tobacco planting soils by metagenomics sequencing.

Previous studies have shown that environmental factors play important roles in shaping microbial community structure and composition^20–22^. Soil pH is found to be an important factor influencing the chemical reactions in soil, and it also affects the availability of soil nutrients impacting plant growth^23^. Notably, pH is reported as the most important factor for determining the microbial community structure in natural environmental systems^24,25^. Our results show that soil amended with wheat straw compost fertiliser maintained pH at 5.7, slightly lower than that with chemical treatment alone (FCK2 pure nitrogen). Although wheat straw compost fertiliser had a relatively small influence on soil pH, a slightly reduced pH relative to other soil treatments may significantly affect soil microbial properties, nutrient availability, root growth, and tobacco plant yields. Previous reports suggest that variation in pH may induce the abundance and composition of acidobacterial community^25,26^. Increased abundances of Acidobacteria versus other bacterial phyla have been observed in more acidic soils^14^. We found Acidobacteria as a predominant phylum and had greater relative abundances in soil treated with wheat straw compost fertilisers.

In addition, variations in soil nutrient properties (e.g. available carbon, total nitrogen, available potassium) commonly cause shifts in soil microbial communities with short-term fertilisation treatments^27–29^. Fertilisation may be a contributing factor favouring a more active, copiotrophic microbial community, thus shifting the predominant microbial life history strategies^17^. Soil organic matter is a critical index of soil fertility due to its capacity to affect plant growth indirectly or directly^30^. Organic fertiliser is a source of organic components^13^, promoting the growth of microbes in soil. Dynamics of soil organic carbon and total nitrogen storage in soils determine microbial activity and nutrient cycles, improves soil physical properties, promotes water retention capacity, and decreases erosion^14^. A great number of genes involved in the function of carbon metabolism were enriched in the associated microbial communities. Proteobacteria, which is known to play critical role in the global cycles of carbon, nitrogen, iron, and sulphur^31–33^, was the most abundant group in soil. Actinobacteria were overrepresented with fertiliser treatments in this study. Actinobacteria are believed to contribute to the global carbon cycle by breaking down plant biomass^28^, and due to their ability to decompose organic matter in soils^34^, they are capable of producing cellulases, hemicellulases, chitinases, glucanases, and amylases^15^.

In line with previous studies showing positive correlations between soil properties and microbial communities, our results identified correlations between soil properties and the dominant phyla. A recent study found that nitrogen, phosphorus, potassium, and combined NPK treatments shifted bacterial community compositions, and under these four fertiliser treatments, Actinobacteria were predominant compared to the control. Redundancy analysis of bacterial community profiles and soil parameters revealed that available trace elements (Mg, total N, Cd, and Al) were positively correlated with variations in community composition^15^.

Furthermore, the application of different amounts of wheat straw compost fertiliser had a certain influence on the number of microbes in the rhizosphere soil of the tobacco field. Communities of bacteria and actinomycetes were highest in the FT1 200 kg/mu straw treatment and the fungal community reached a peak in the FT2 300 kg/mu straw treatment. The communities of bacteria and fungi reached a peak at 60 days post-transplantation whereas the actinomycete community reached its peak at 90 days. These microbial delineations were positively correlated with the amount of wheat straw compost fertiliser. The quality of the soil surrounding the root is dependent on the organic material from the vicinity of root and the microbial activity on nutrient cycling and plant growth. The composition of bacteria and fungi populations has positive, negative, or neutral role on plants depending on the soil conditions^35^. One goal of fertilisation management is to increase the beneficial effects of soil microbial community. However, the association between fertilisation and the population of bacteria and fungi has not been determined. Further analyses are urgently needed.

The application of different wheat straw compost fertiliser concentrations had a certain influence on the economic traits of tobacco. With different applications of wheat straw compost fertiliser, the content of total sugars, reducing sugars, chlorine and potassium in roots and leaves and the content of nicotine in root system increased to certain extent. The increased total sugars and reducing sugars under the use of wheat straw compost fertiliser treatments did improve the performance of flue-cured samples. High levels of chlorine have been shown to affect the flammability of tobacco, while high levels of potassium can lead to a lack of coordination between potassium and chlorine^36–38^. In the present study, nicotine and potassium contents were not affected significantly but increased applications of wheat straw compost fertiliser did lead to an increase in chlorine content. It is reported that microbial metabolism is responsible for the fate of chemical components in the biosphere^39^. β-Proteobacterium has been found to be capable of degradation of chlorides and provide a critical link in the chain of microbial metabolism^40^. In this study, Proteobacteria was found to have high abundance in straw fertiliser treated soil, which may contribute to the increase of chlorine content in soils. Overall, the application of FT1 200 kg/mu straw showed a comprehensive superiority in the overall desirable tobacco traits. We speculated that the dominated microbial populations play key roles in the variety of soil properties. Therefore, to improve tobacco quality and its economic qualities, growers need to consider applying an appropriate amount of wheat straw compost fertiliser during growth.

## Conclusion

In conclusion, chemical fertiliser combined with wheat straw compost fertiliser improved soil fertility, tobacco quality, and economic traits. It also significantly shifted the microbial community structure. A significant correlation was identified between soil properties and the dominant phyla under different fertilisation treatments in our study. The chemical fertiliser combined with straw compost fertiliser can effectively improve the soil traits and microbial diversity. The straw compost fertiliser could be widely used in agricultural production. The application of straw compost fertiliser may cure soil contamination induced by chemical products.

## Materials and Methods

### Fertiliser treatments

**Figure 7:**
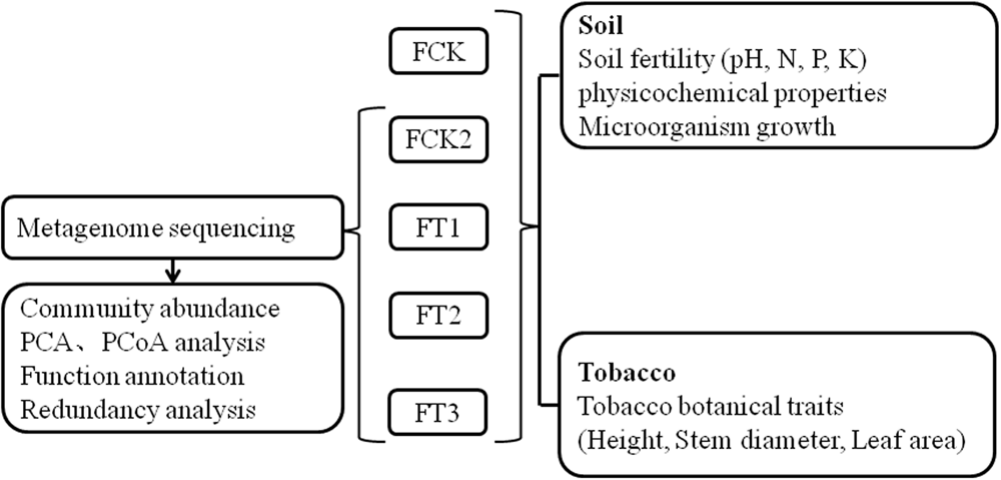
The experimental design.

The experiment was carried out in 2015 in a planting field growing Zhongyan-100 tobacco variety in Tongchen Village, WenfengTwon, Wuyang County, Luohe City, Henan Province, China. The medium fertile soil contained 22.22 mg/kg soil organic matter, 124.01 mg/kg available nitrogen (N), 54.54 mg/kg available phosphorus (P), 108.06 mg/kg available potassium (K) and pH 5.31. Fertiliser treatments included: 1) nofertiliser application (CK), 2) 2.5 kg/mu pure nitrogen fertiliser (FCK2 pure nitrogen), 3) 2.5 kg/mu pure nitrogen fertiliser with 200 kg/mu straw compost (FT1 200 kg/mu straw), 4) 2.5 kg/mu pure nitrogen fertiliser with 300 kg/mu straw compost (FT2 300 kg/mu straw), and 5) 2.5 kg/mu pure nitrogen fertiliser with 400 kg/mu straw compost (FT3 400 kg/mu straw). Pure nitrogen fertiliser was 2.5 kg/mu with ratios of N:P_2_O_5_:K_2_O = 1:1:3.5. A 70% dilution of fertiliser was used as base fertiliser during ridging, and a 30% dilution was applied during transplantation. Transplant spacing was 120 cm × 50 cm. The wheat straw was crushed and decomposed with straw decomposer for 18–22 days before applied to the field. The decomposed straw was evenly applied to the test field and soils were prepared by rotary tillage, watering, and sedimentation for one week followed by repeated tillage. The randomised block plots with sizes of 60–80 m^2^ were set with 6–8 lines in each block and two protection lines between blocks. For metagenomic sequencing, soil samples from FCK2 and wheat straw compost fertilisers (FT1 200 kg/mu straw, FT2 300 kg/mu straw and FT3 400 kg/mu straw) were used. A major flow in the experimental design is shown in Fig. 7.

### Tobacco traits and properties assessments

Agronomic traits and chemical composition of the tobacco were assessed 30, 45, 60, 75, 90, and 105 days post-transplantation. AA3 Continuous Flow Analytical System was used to determine chemical composition of tobacco, including nicotine, chloride, potassium, and total/reducing sugars, and ICP was used to detect macro-/micro-elements.

### Soil samples collection

Five fertiliser treatments were repeated 3 times and the rhizosphere soils were collected at a depth of 5–40 cm at 30, 45, 60, 75, 90 and 105 days post-transplantation with five cores for each. Three repeated samples for each point were mixed equally and sieved. Then each soil sample was divided into two parts, one of which was subjected to biochemical analysis while the other was used for DNA extraction for metagenome sequencing. Sub-samples were frozen at −80 °C.

### Analysis pf soil properties

Soil samples were subjected to chemical analysis. Soil pH was checked by pH-electrometric titration. Soil organic matter content and available N were measured by potassium dichromate external heating method and alkali-hydrolysed reduction diffusing method. NaHCO_3_ colorimetric method was used to determine soil available P and soil available K extracted with NH_4_OAc and measured by flame photometry method. For bacterial cultures, beef extract-peptone agar (0.3 g of beef extract, 1 g of peptone, 0.5 g of NaCl, 2 g of agar and 100 g of distilled water) was used. Fungus were cultured on Martin selective medium (0.1 g of KH_2_PO_4_·3H_2_O, 0.05 g of MgSO_4_·7H_2_O, 0.33 ml of 0.1% Bangladesh red solution, and 100 ml distilled water). Actinomycete cultures were grown using improved Gauze’s no. 1 medium (0.1 g of KNO_3_, 0.05 g of K_2_HPO_4_, 0.05 g of MgSO_4_·7H_2_O, 0.05 g of NaCl and 0.001 g of FeSO_4_· 7H_2_O). After serial dilutions and plating, soil microbial colonies were counted.

### DNA extraction and metagenomic sequencing

Fresh soil samples were used for DNA extraction with the E.Z.N.A. Soil DNA kit (OMEGA Bio-Tek, Inc., Norcross, GA, USA). DNA library was constructed using TruSeq™ DNA Sample Prep Kit and subjected to Illumina Hiseq sequencing (OE Biotech CO., LTD, Shanghai, China). In detail, genomic DNA was checked by 1% agarose gel electrophoresis at various steps like after fragmentation, end-repair for DNA samples with ultrasonic treatment, A-tailing, and addition of sequencing adapters to both end of fragments. After removal of self-ligated fragments by magnetic beads purification and fragment screening, library amplification was performed by PCR. For sequencing data, reads containing less than 70% bases with Q20 were filtered out using NGSQC toolkit (version 2.3.2)^41^. Metagenome assembly was performed using SOAPdenovo2 (version 2.0)^42^ after getting valid reads. Gaps inside the scaffold were used as breakpoint to interrupt the scaffold into new contigs (ScafContig) and these new contigs with length >/= 200 bp were retained. ORF prediction of assembled scaffolds using prodigal (version 2.6.3)^43^ was performed and translated into amino acid sequences. The non-redundant gene sets were built for all predicted genes using CDHIT (version 4.5.4)^44^. The clustering parameters included 95% identity and 90% coverage. The longest gene was selected as representative sequence of each gene set.

Clean reads of each sample were aligned against the non-redundant gene set (95% identity) use (version 2.1.0)^45^, and the abundant information of the gene in the corresponding sample was counted. The gene set representative sequence (amino acid sequence) was annotated with NR, KEGG, COG, SWISSPROT, and GO database with an e-value of 1e-5. The taxonomy of the species was studied as a result of the corresponding taxonomy database of the NR Library and the abundance of the species was calculated using the corresponding abundance of the genes. In order to construct the abundance profile on the corresponding taxonomy level, abundance statistics were performed at each level of Domain, Kingdom, Phylum, Class, Order, Family, Genus, Species. The GeneMark.hmm (version 3.26) in GeneMark software were used to predict the potential protein coding region in a DNA sequence. Representative sequences for enriched genes were used to label the drug resistance genes. The gene sets were compared with the CAZy database using the corresponding tool hmmscan (version 3.1b2) to obtain the information of the carbohydrate active enzyme corresponding to the gene and then calculated the carbohydrate activity using the sum of the gene abundances corresponding to the carbohydrate active enzyme abundance.

The PCA analysis and plotting of the abundance spectrum of the species or functional abundance spectrum were carried out using R software (version 3.2.0), and the results of the equidistant matrix of PCoA and NMDS were calculated and analysed.

### Correlation between soil properties and microbial communities

Redundancy analysis is an alternative analysis of Canonical Correlation Analysis. In order to analyse the relationship between microbial populations and soil properties, the redundancy analysis was performed by vegan package in R.

### Statistical analysis

Statistical analysis was performed using SPSS 18.0. The differences among groups were analysed by ANOVA (Analysis of variance) and the comparison between groups was performed by LSD (least significant difference) method. All the differences with p-value < 0.05 were considered as significant differences.

## Supplementary information

Supplementary files
