# Supplementary material for: Metagenomic insights into effects of wheat straw compost fertiliser application on microbial community composition and function in tobacco rhizosphere soil
Source: Sci Rep. 2019 Apr 16;9:6168. doi: 10.1038/s41598-019-42667-z (PMC6467887; doi:10.1038/s41598-019-42667-z)
Supplement: Supplementary file 1 — Supplementary files [file 41598_2019_42667_MOESM1_ESM.docx]

**Metagenomic insights into effects of** **wheat straw compost fertiliser application on microbial community composition and function in tobacco rhizosphere soil**

Yong-feng Yang^a#^, Song-jie zhang^a#^, Ning Li^a^, Hong-Li Chen^a^, Hong-fang Jia^a^, Xiao-ning Song^a^, Guo-shun Liu^a^, Chao Ni^b^, Zhi-zhong Wang^c^, Hui-fang Shao^a^ and Song-tao Zhang^a^*

*^a^ Henan Agricultural University, College of Tobacco Science, Tobacco Cultivation Key Laboratory of China Tobacco, Zhengzhou 450002, China*

*^b^ China Tobacco Chongqing Industrial Corporation, Chongqing 400000, China*

*^c^ Wuyang County Tobacco Branch of Luohe Tobacco Company, Luohe, Henan Province 462000, China*

**^#^These authors contribute equally to the work**

***Corresponding Author:**

E-mail address: Song-tao Zhang, zhangsongzi@163.com (*Correspondence and reprints)

**Supplementary files:**

**Figure S1**. Heatmap of soil microbial community abundance profiles at the family level.


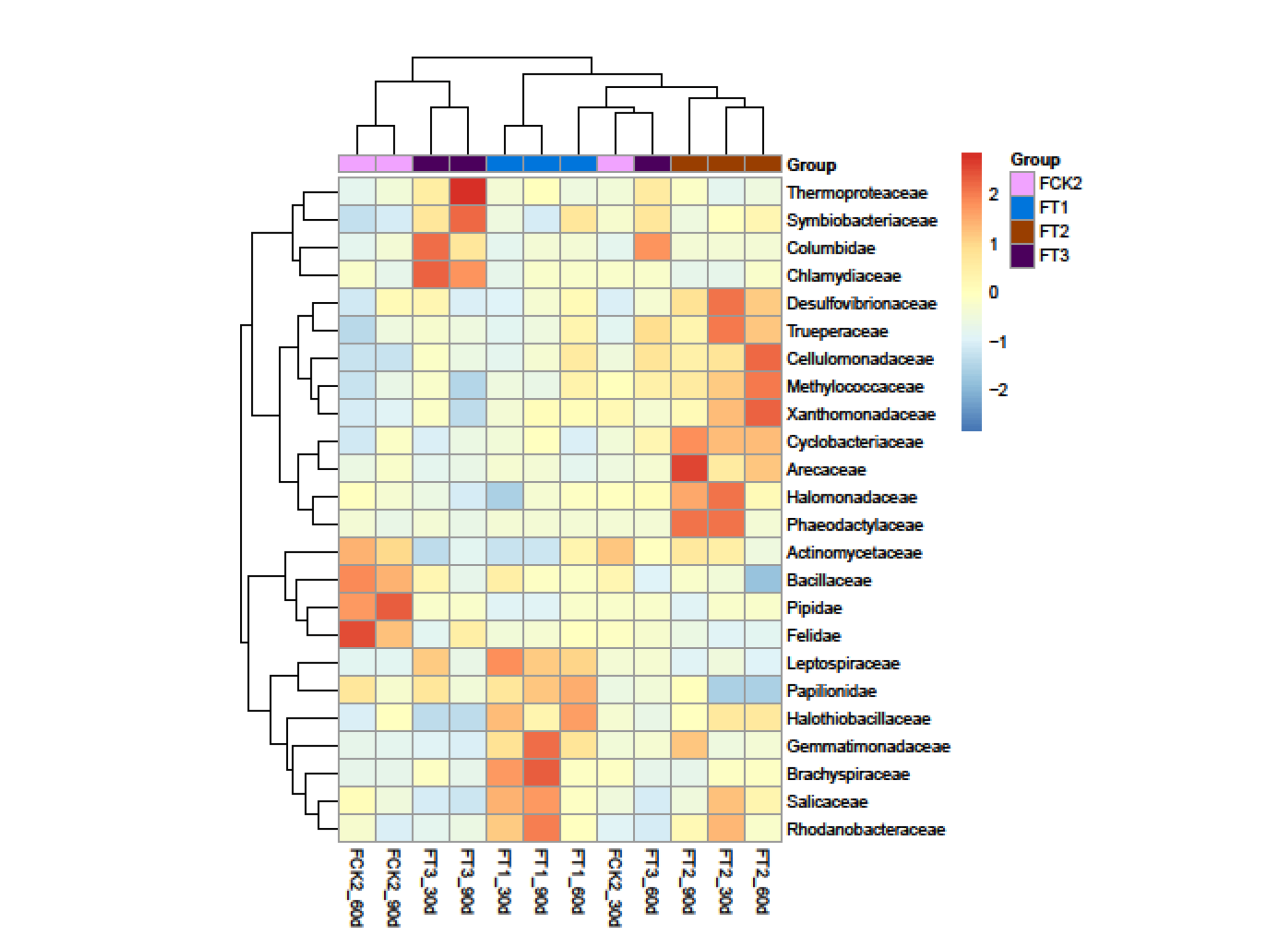


**Figure S2**. Heatmap of soil microbial community abundance profiles for a taxonomic comparison of the sampled communities under the different fertilization treatments at the phyla level (a) and the order level (b).


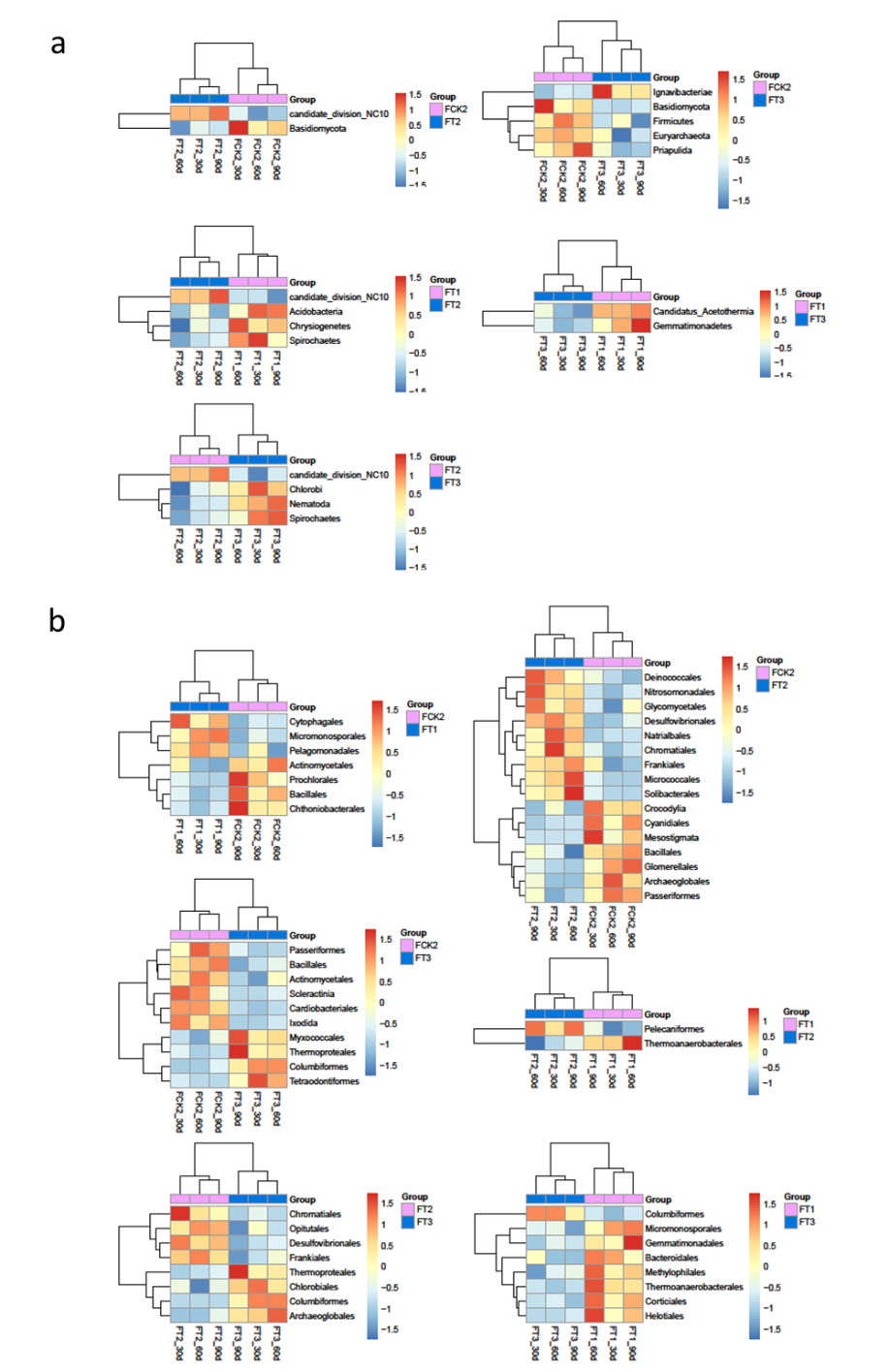


**Figure S3**. Heatmap of Kyoto Encyclopedia of Genes and Genomes (KEGG) pathways.


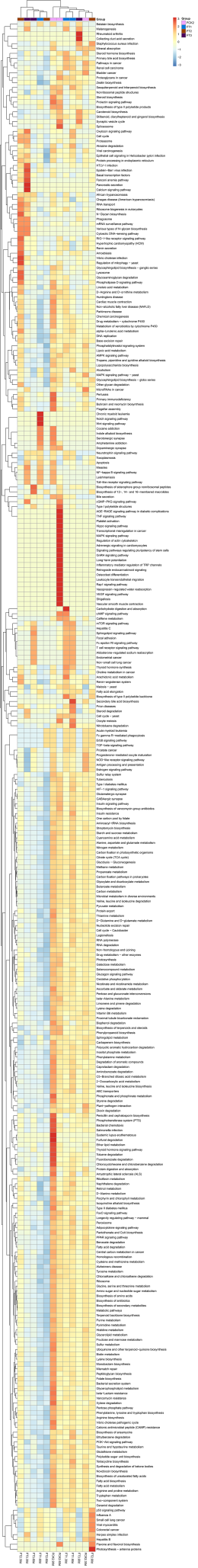


Table S1. Total sugar content of tobacco under different fertilizer treatments (%)

| Category | Treatment | Days after transplanting/d | | | | | |
| --- | --- | --- | --- | --- | --- | --- | --- |
|  |  | 50 | | 60 | | 90 | |
| Root | FCK | 1.05 | c | 1.04 | b | 6.25 | b |
|  | FCK2 | 1.94 | b | 1.17 | b | 9.94 | a |
|  | FT1 | 1.75 | b | 0.96 | b | 10.17 | a |
|  | FT2 | 1.82 | b | 1.88 | a | 9.74 | a |
|  | FT3 | 2.65 | a | 1.22 | b | 6.58 | b |
|  |  |  |  |  |  |  |  |
| Stem | FCK | 11.88 | a | 7.84 | a | 7.00 | ab |
|  | FCK2 | 14.49 | a | 3.35 | a | 12.26 | a |
|  | FT1 | 15.87 | a | 6.57 | a | 6.69 | ab |
|  | FT2 | 13.31 | a | 4.75 | a | 3.20 | b |
|  | FT3 | 15.31 | a | 8.28 | a | 6.21 | b |
|  |  |  |  |  |  |  |  |
| Leaf | FCK | 4.57 | c | 1.49 | a | 3.35 | b |
|  | FCK2 | 5.87 | bc | 2.82 | a | 9.57 | a |
|  | FT1 | 6.13 | b | 3.15 | a | 7.78 | ab |
|  | FT2 | 4.75 | c | 3.39 | a | 6.74 | ab |
|  | FT3 | 7.45 | a | 3.02 | a | 8.24 | ab |

Note: Lowercase letters show significant difference at 5% level.

Table S2. Reducing sugar content of tobacco under different fertilizer treatment (%)

| Category | Treatment | Days after transplanting/d | | | | | |
| --- | --- | --- | --- | --- | --- | --- | --- |
|  |  | 50 | | 60 | | 90 | |
| Root | FCK | 0.76 | d | 0.55 | a | 3.29 | a |
|  | FCK2 | 1.42 | ab | 0.58 | a | 4.03 | a |
|  | FT1 | 1.32 | bc | 0.59 | a | 4.78 | a |
|  | FT2 | 1.07 | c | 0.93 | a | 4.36 | a |
|  | FT3 | 1.69 | a | 0.57 | a | 3.55 | a |
|  |  |  |  |  |  |  |  |
| Stem | FCK | 11.37 | a | 6.08 | ab | 4.56 | b |
|  | FCK2 | 13.40 | a | 2.47 | b | 10.12 | a |
|  | FT1 | 14.76 | a | 5.52 | ab | 5.13 | b |
|  | FT2 | 12.40 | a | 3.75 | ab | 1.76 | b |
|  | FT3 | 15.01 | a | 7.09 | a | 4.12 | b |
|  |  |  |  |  |  |  |  |
| Leaf | FCK | 3.99 | b | 0.59 | a | 2.12 | b |
|  | FCK2 | 6.06 | a | 1.96 | a | 8.38 | a |
|  | FT1 | 5.54 | a | 2.36 | a | 6.83 | ab |
|  | FT2 | 3.85 | b | 2.72 | a | 6.02 | ab |
|  | FT3 | 6.77 | a | 2.34 | a | 5.77 | ab |

Note: Lowercase letters show significant difference at 5% level.

Table S3. Nicotine content of tobacco under different fertilizer treatments (%)

| Category | Treatment | Days after transplanting/d | | | | | |
| --- | --- | --- | --- | --- | --- | --- | --- |
|  |  | 50 | | 60 | | 90 | |
| Root | FCK | 0.11 | d | 0.12 | a | 0.45 | ab |
|  | FCK2 | 0.14 | cd | 0.11 | a | 0.55 | ab |
|  | FT1 | 0.16 | bc | 0.09 | a | 0.57 | a |
|  | FT2 | 0.19 | b | 0.12 | a | 0.54 | ab |
|  | FT3 | 0.23 | a | 0.11 | a | 0.42 | b |
|  |  |  |  |  |  |  |  |
| Stem | FCK | 0.16 | a | 0.23 | a | 0.26 | a |
|  | FCK2 | 0.20 | a | 0.23 | a | 0.23 | ab |
|  | FT1 | 0.15 | a | 0.30 | a | 0.18 | b |
|  | FT2 | 0.15 | a | 0.28 | a | 0.16 | b |
|  | FT3 | 0.14 | a | 0.29 | a | 0.19 | b |
|  |  |  |  |  |  |  |  |
| Leaf | FCK | 0.52 | a | 0.07 | a | 1.41 | a |
|  | FCK2 | 0.39 | b | 0.05 | ab | 1.28 | a |
|  | FT1 | 0.46 | ab | 0.04 | b | 1.15 | a |
|  | FT2 | 0.44 | b | 0.06 | ab | 1.51 | a |
|  | FT3 | 0.39 | b | 0.05 | ab | 1.49 | a |

Note: Lowercase letters show significant difference at 5% level.

Table S4. Chloride content of tobacco under different fertilizer treatments (%)

| Category | Treatment | Days after transplanting/d | | | | | |
| --- | --- | --- | --- | --- | --- | --- | --- |
|  |  | 50 | | 60 | | 90 | |
| Root | FCK | 0.13 | c | 0.41 | a | 0.06 | c |
|  | FCK2 | 0.21 | c | 0.23 | b | 0.08 | c |
|  | FT1 | 0.34 | b | 0.19 | b | 0.16 | b |
|  | FT2 | 0.52 | a | 0.45 | a | 0.15 | b |
|  | FT3 | 0.53 | a | 0.31 | ab | 0.23 | a |
|  |  |  |  |  |  |  |  |
| Stem | FCK | 0.59 | d | 0.68 | ab | 0.50 | c |
|  | FCK2 | 0.74 | c | 0.80 | ab | 0.54 | c |
|  | FT1 | 1.10 | b | 0.97 | a | 0.69 | bc |
|  | FT2 | 1.25 | a | 0.46 | b | 0.87 | b |
|  | FT3 | 1.22 | a | 0.78 | ab | 1.15 | a |
|  |  |  |  |  |  |  |  |
| Leaf | FCK | 0.37 | c | 0.85 | a | 0.58 | d |
|  | FCK2 | 0.59 | c | 0.94 | a | 1.18 | c |
|  | FT1 | 1.25 | b | 0.90 | a | 1.78 | b |
|  | FT2 | 1.69 | a | 0.60 | a | 2.17 | a |
|  | FT3 | 1.53 | ab | 0.63 | a | 1.75 | b |

Note: Lowercase letters show significant difference at 5% level.

Table S5. Potassium content of tobacco under different fertilizer treatments (%)

| Category | Treatment | Days after transplanting/d | | | | | |
| --- | --- | --- | --- | --- | --- | --- | --- |
|  |  | 50 | | 60 | | 90 | |
| Root | FCK | 1.65 | bc | 2.12 | a | 1.91 | a |
|  | FCK2 | 1.52 | c | 1.94 | a | 1.54 | b |
|  | FT1 | 1.49 | c | 1.90 | a | 1.55 | b |
|  | FT2 | 2.01 | a | 1.77 | a | 1.55 | b |
|  | FT3 | 1.8841 | ab | 1.72 | a | 1.69 | b |
|  |  |  |  |  |  |  |  |
| Stem | FCK | 4.79 | a | 3.58 | bc | 3.37 | a |
|  | FCK2 | 4.14 | ab | 4.13 | a | 2.26 | b |
|  | FT1 | 3.99 | b | 3.40 | c | 2.53 | b |
|  | FT2 | 3.73 | b | 3.88 | ab | 2.50 | b |
|  | FT3 | 3.82 | b | 2.91 | d | 2.77 | b |
|  |  |  |  |  |  |  |  |
| Leaf | FCK | 3.57 | a | 3.98 | a | 4.39 | a |
|  | FCK2 | 3.05 | a | 3.90 | ab | 2.99 | b |
|  | FT1 | 2.80 | a | 3.46 | c | 2.39 | bc |
|  | FT2 | 2.91 | a | 3.70 | abc | 2.36 | c |
|  | FT3 | 2.72 | a | 3.50 | bc | 2.44 | bc |

Table S6. Summary of functional annotations against NR, SWISSPROT, KEGG, COG and GO databases.

| Sample | Stat | NR | SWISSPROT | KOG | KEGG | GO |
| --- | --- | --- | --- | --- | --- | --- |
| Sample_FCK2_30d | annotation_numbers | 122229 | 66812 | 107329 | 23347 | 64829 |
| Sample_FCK2_30d | annotation_ratio | 78.06% | 42.67% | 68.55% | 14.91% | 41.40% |
| Sample_FCK2_60d | annotation_numbers | 138739 | 82695 | 134953 | 21827 | 80209 |
| Sample_FCK2_60d | annotation_ratio | 66.66% | 39.73% | 64.84% | 10.49% | 38.54% |
| Sample_FCK2_90d | annotation_numbers | 116287 | 81314 | 131948 | 30178 | 78915 |
| Sample_FCK2_90d | annotation_ratio | 55.11% | 38.54% | 62.53% | 14.30% | 37.40% |
| Sample_FT1_30d | annotation_numbers | 90386 | 52036 | 81829 | 20941 | 50500 |
| Sample_FT1_30d | annotation_ratio | 70.54% | 40.61% | 63.87% | 16.34% | 39.41% |
| Sample_FT1_60d | annotation_numbers | 62292 | 40909 | 68288 | 9924 | 39727 |
| Sample_FT1_60d | annotation_ratio | 57.27% | 37.61% | 62.78% | 9.12% | 36.52% |
| Sample_FT1_90d | annotation_numbers | 165652 | 88914 | 144161 | 24640 | 86148 |
| Sample_FT1_90d | annotation_ratio | 75.59% | 40.57% | 65.79% | 11.24% | 39.31% |
| Sample_FT2_30d | annotation_numbers | 62520 | 39128 | 61929 | 18438 | 37934 |
| Sample_FT2_30d | annotation_ratio | 69.42% | 43.44% | 68.76% | 20.47% | 42.12% |
| Sample_FT2_60d | annotation_numbers | 40487 | 21123 | 34495 | 8114 | 20568 |
| Sample_FT2_60d | annotation_ratio | 75.94% | 39.62% | 64.70% | 15.22% | 38.58% |
| Sample_FT2_90d | annotation_numbers | 132234 | 71812 | 119190 | 19411 | 69595 |
| Sample_FT2_90d | annotation_ratio | 72.14% | 39.18% | 65.02% | 10.59% | 37.97% |
| Sample_FT3_30d | annotation_numbers | 70294 | 37978 | 61941 | 13235 | 36808 |
| Sample_FT3_30d | annotation_ratio | 72.77% | 39.32% | 64.12% | 13.70% | 38.10% |
| Sample_FT3_60d | annotation_numbers | 75500 | 40550 | 67215 | 13128 | 39416 |
| Sample_FT3_60d | annotation_ratio | 71.73% | 38.53% | 63.86% | 12.47% | 37.45% |
| Sample_FT3_90d | annotation_numbers | 126400 | 69774 | 114465 | 18714 | 67664 |
| Sample_FT3_90d | annotation_ratio | 72.84% | 40.21% | 65.96% | 10.78% | 38.99% |

Table S7. Taxonomic classification annotations.

| Taxon_level | Archaea | Bacteria | Eukaryota | Viruses | Unclassified organisms |
| --- | --- | --- | --- | --- | --- |
| Sample_FCK2_30d | 3.1% | 68.7% | 21.1% | 6.3% | 0.8% |
| Sample_FCK2_60d | 3.0% | 64.1% | 20.7% | 11.3% | 0.9% |
| Sample_FCK2_90d | 3.6% | 65.8% | 15.8% | 14.2% | 0.7% |
| Sample_FT1_30d | 1.4% | 65.7% | 24.9% | 7.5% | 0.5% |
| Sample_FT1_60d | 2.9% | 78.5% | 14.1% | 3.8% | 0.6% |
| Sample_FT1_90d | 4.7% | 72.2% | 19.0% | 3.0% | 1.0% |
| Sample_FT2_30d | 1.8% | 70.8% | 21.4% | 5.8% | 0.1% |
| Sample_FT2_60d | 1.2% | 72.7% | 17.1% | 8.4% | 0.6% |
| Sample_FT2_90d | 4.2% | 70.9% | 19.4% | 4.7% | 0.8% |
| Sample_FT3_30d | 2.4% | 65.3% | 26.9% | 5.2% | 0.3% |
| Sample_FT3_60d | 2.9% | 64.6% | 20.5% | 11.6% | 0.4% |
| Sample_FT3_90d | 3.5% | 75.9% | 19.1% | 0.9% | 0.6% |
